# Development of Pigmentation-Regulating Agents by Drug Repositioning

**DOI:** 10.3390/ijms22083894

**Published:** 2021-04-09

**Authors:** Seo-Mi-Gon Jeong, Tae-Jin Yoon

**Affiliations:** 1 Department of Dermatology, School of Medicine, Gyeongsang National University & Hospital, Jinju 52727, Korea; goniing@naver.com; 2 Institute of Health Sciences, School of Medicine, Gyeongsang National University & Hospital, Jinju 52727, Korea

**Keywords:** drug repositioning, melanogenesis, signaling pathways

## Abstract

Skin color is determined by the processes of melanin synthesis and distribution. Problems in various molecules or signaling pathways involved in melanin synthesis contribute to skin pigmentation defects. Several trials have been conducted on the production of pigmentation-regulating agents, and drug repositioning has emerged as a modern technique to identify new uses for existing drugs. Our research team has researched substances or drugs associated with pigmentation control and, as a result, nilotinib, sorafenib, and ICG-001 have been found to promote pigmentation, while 5-iodotubercidin inhibits pigmentation. Therefore, these substances or medications were suggested as potential therapeutics for pigmentation disorders by drug repositioning.

## 1. Introduction

Melanocytes are cells that play an important role in expressing skin color, with a particular function in the synthesis and distribution of melanin. Melanin synthesis occurs in melanosomes, specialized membrane-bound organelles inside melanocytes. Each melanocyte is linked to several keratinocytes, and melanin pigments are continuously distributed to the surroundings [1]. Several studies on melanocyte growth and differentiation have been performed, and the regulation of melanin synthesis is known to include a variety of molecules and intracellular signaling pathways. Abnormalities in the melanin production and development processes contribute to skin pigmentation abnormalities, such as hypopigmentation (whitening) or hyperpigmentation (darkening). Since these pigmentation disorders have a negative effect on the quality of life, researchers in dermatology and cosmetics have concentrated on the development of pigmentation-regulating agents from various sources [2,3]. Since studies on pigmentation-regulating agents often produce new medicines from herbal extracts or chemical compounds, it is thought that the direct application in actual clinical trials relative to the time and expense of development will be impractical. Despite the considerable concern and investment in the production of new medicine, some could not be adequately used for existing indications owing to the lack of effects or side effects arising in the preclinical or clinical stages. Drug repositioning is now emerging as a new approach to rediscovering new indications of drugs already approved, rather than starting at the beginning stage of drug production [4].

Drug development by drug repositioning has been in progress in various fields in recent years but has not been adequately implemented in fields related to pigmentation-regulating agents. For several years, our research team has been undertaking experiments to identify substances or drugs involved in the pigmentation process. We investigated 800 drugs approved by the United States (US) Food and Drug Administration (FDA), and in vitro or in vivo experiments were conducted. In the in vitro experiments, pigmented cell lines, promoter assays, and normal human melanocytes were used, and in the in vivo experiments, zebrafish were used. It is thought that the probability of identifying compounds for therapeutic use, which is proved from studies of novel mechanisms involved in the regulation of pigmentation in addition to the already known effects of drugs, is strong. This review briefly identifies the mechanisms of melanin synthesis and development to explain the etiology of associated pigmentation disorders, and discusses the use of drug repositioning as a potential development strategy for pigmentation-regulating agents. Specifically, it presents a review of the findings and interpretations of several studies on pigmentation-regulating substances and drugs undertaken by our research team.

## 2. Materials and Methods

### 2.1. Search Strategy

For this review, we used the adapted preferred reporting items for systematic reviews and meta-analyses (PRISMA) model. For the literature search, we used an electronic database search in PubMed and Scopus (first search in November 2020; updated in March 2021). We searched these databases using the following search terms: “pigmentation”, “melanogenesis” OR “drug repositioning”.

### 2.2. Inclusion and Exclusion Criteria

We scanned all articles included in the research field. We included English articles referring to drug repositioning in melanogenesis. Due to the small number of search results, we did not apply filters regarding the year of publication or type of study. Articles that analyzed the general mechanisms of regulating melanogenesis, the concept of drug repositioning and examples of applications in dermatology were included. We included our previous research on the development of pigmentation-regulating agents by drug repositioning. We excluded articles in which the mechanisms were not related to melanogenesis or in which there was no mention of drug repositioning.

### 2.3. Study Selection

**Figure 1:**
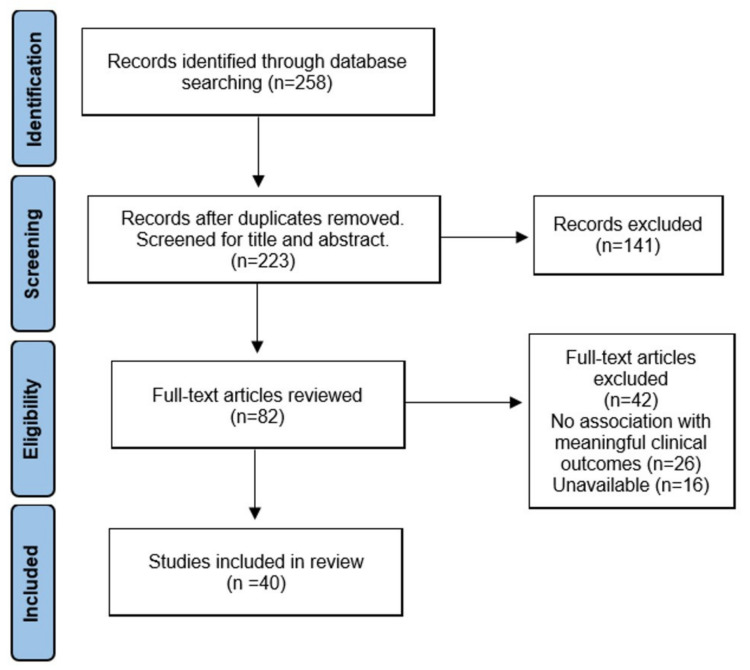
The preferred reporting items for systematic reviews and meta-analyses (PRISMA) flow diagram.

Of a total of 258 articles, 35 were duplicates and were removed. The title and abstract of 223 articles were screened, and 82 articles were reviewed in full text. Subsequently, full-text assessment resulted in the exclusion of 42 studies in total, including 26 studies that were not associated with clinical outcomes and 16 studies that did not have the full text available. In the end, 40 studies were included in this review. A flowchart for study selection is depicted in Figure 1.

## 3. Signaling Pathways in the Regulation of Melanogenesis

**Figure 2:**
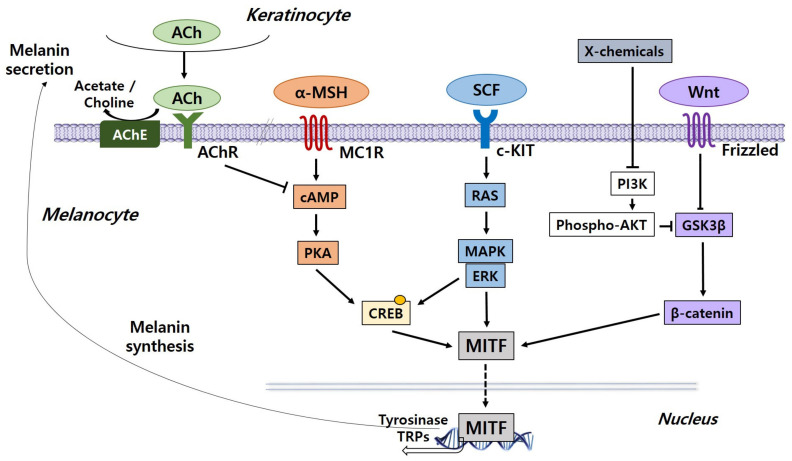
Core signaling pathways associated with the regulation of melanogenesis in melanocytes. Genes encoding specific melanogenic enzymes, including tyrosinase and TRPs, are regulated by the MITF transcription factor, which is consequently regulated by a number of signaling pathways, including α-MSH/MC1R (orange), SCF/KIT (blue) and Wnt/frizzled (purple). Signal transduction is mediated by cAMP/PKA, RAS/MAPK and β catenin pathways. The cholinergic system is a negative regulator of melanogenesis. ACh, acetylcholine; AChE, acetylcholinesterase; AChR, acetylcholine receptor; α-MSH, α-melanocyte-stimulating hormone; cAMP, cyclic adenosine monophosphate; CREB, cAMP response element-binding protein; ERK, extracellular signal-regulated protein kinase; GSK3β, glycogen synthase kinase 3β; c-KIT, tyrosine kinase receptor; MAPK, mitogen-activated protein kinase; MC1R, melanocyte-specific melanocortin 1 receptor; MITF, microphthalmia-associated transcription factor; PKA, protein kinase A; PI3K, phosphatidylinositol 3-kinase; SCF, stem cell factor; TRPs, tyrosinase-related proteins; Wnt, wingless-related integration site.

Melanogenesis is a complicated process mediated by multiple signaling pathways, and microphthalmia-associated transcription factor (MITF), which exists downstream of these pathways, is emphasized as a major regulator of melanocyte production. Transcription of the MITF gene is regulated by major signaling pathways including α-melanocyte-stimulating hormone (α-MSH)/melanocortin 1 receptor (MC1R), wingless-related integration site (Wnt)/β-catenin, and stem cell factor (SCF)/tyrosine kinase receptor (KIT) pathways. Problems in these signaling pathways can lead to defects in skin pigmentation such as hypopigmentation and hyperpigmentation. We discuss the regulation of melanogenesis by several signaling pathways [5] (Figure 2).

### 3.1. The α-MSH/MC1R Signaling Pathway

The melanogenic inducer α-MSH combines with the MC1R on melanocytes to activate intracellular cyclic adenosine monophosphate (cAMP). The effect of increased cAMP is regulated by cAMP-dependent protein kinase A (PKA). This protein triggers the phosphorylation of the cAMP response element-binding protein (CREB), which promotes the gathering of the CREB-binding protein (CBP). Pigmentation-related gene expression, including MITF, tyrosinase, and tyrosinase-related proteins (TRPs), is subsequently increased [6,7].

### 3.2. Wnt/β-Catenin Signaling Pathway

Another regulatory process for melanocyte development is the Wnt/β-catenin signaling pathway. As Wnt binds to the receptors, the stability of cytoplasmic β-catenin increases and activates MITF transcription in the nucleus [5]. The amount of cytoplasmic β-catenin is considered to be regulated by the destruction complex of various molecules such as adenomatous polyposis coli (APC), glycogen synthase kinase 3β (GSK3β), and axin. GSK3β phosphorylates β-catenin without Wnt signaling, leading to the decomposition of phosphorylated β-catenin through a proteasome-dependent process. Phosphorylation of serine 9 (S9) of GSK3β reduces its enzyme activity, thereby inhibiting β-catenin phosphorylation and melanogenesis [8]. Phosphorylation of tyrosine 216 (Y216) of GSK3β blocks the recruitment of phosphorylated β-catenin to E3 ligase β-TrCP, activating β-catenin signaling and melanogenesis [9]. In brief, overexpressed β-catenin via Wnt signaling stimulates the expression of MITF, a vital component of the growth of melanocytes, contributing to an increase in pigmentation.

### 3.3. SCF/KIT Signaling Pathway

In melanogenesis, the SCF/KIT signaling pathway is also important. When SCF interacts with c-KIT, its receptor, tyrosine kinase activity is stimulated, triggering receptor phosphorylation. The phosphorylation of c-KIT involves the stimulation of mitogen-activated protein kinase (MAPK) and CREB phosphorylation occurs, activating MITF. Thus, inhibiting the SCF/KIT signaling pathway can decrease melanin pigmentation [5].

### 3.4. Endothelin Signaling Pathway

In the process of ultraviolet B (UVB)-induced pigmentation, endothelin (ET), which is formed in keratinocytes, is a mediator for melanocytes. Exposure to ultraviolet B (UVB) rays induces the connection of ET between melanocytes and keratinocytes. ET increases tyrosinase activity and enhances gene expression, including tyrosinase and tyrosinase-related protein-1 (TRP-1) expression [10,11].

### 3.5. Acetylcholine Signaling Pathway

The cholinergic system may be a negative regulator of skin pigmentation. The release of acetylcholine (ACh) from keratinocytes is activated by solar light. ACh binds to the acetylcholine receptor (AChR) and acetylcholinesterase (AChE) of melanocytes, resulting in the inhibition of the cAMP-dependent signaling pathway. Melanogenesis caused by solar light in melanocytes is suppressed by ACh and AChE inhibitors, indicating that the cholinergic mechanism plays a part in inhibiting melanogenesis [12,13].

### 3.6. Phosphatidylinositol 3-Kinase/AKT Signaling Pathway

Phosphatidylinositol 3-kinase (PI3K)/protein kinase B (AKT) signaling, which prevents the process of pigmentation, is another mechanism. The activation of AKT signaling suppresses GSK3β and blocks pigmentation, resulting in a decrease in the melanin content. On the other hand, the inhibition of PI3K and downstream AKT signaling promotes GSK3β activation, facilitating the process of MITF attaching to the promoter of tyrosinase. Consequently, the expression of pigmentation-associated genes is enhanced [14].

### 3.7. Extracellular Signal-Regulated Protein Kinase Signaling Pathway

The extracellular signal-regulated protein kinase (ERK) signaling pathway is activated by c-KIT, and also promotes the phosphorylation of CREB. Activated ERK signaling also stimulates phosphorylation and causes the degradation of MITF, which prevents the synthesis of melanin [5,15].

## 4. Drug Repositioning

### 4.1. Concept and Advantage of Drug Repositioning

The production of a new drug involves complicated stages, including discovery and preclinical stages, safety review, clinical research, FDA review, and FDA post-market safety monitoring [16]. Several issues are associated with these conventional drug production methods, such as enormous costs, lengthy developmental times, and insufficient test findings on drug side effects or toxicity. Drug repositioning seeks new uses by re-evaluating existing drugs already licensed. Drug repositioning is viewed as an alternative technique because of many benefits over the current manufacturing of drugs. It decreases time and expenses since there are only four phases: compound identification, compound acquisition, development, and FDA post-market safety monitoring [16]. There is no need to conduct the initial synthesis or optimization required to discover new drugs, as existing drugs have passed through the preclinical and clinical stages. Furthermore, evidence on the safety or toxicity of the drugs has also been investigated. Therefore, there are benefits in that the expense and time needed to produce new drugs are greatly reduced and the risk of production failure due to safety concerns is reduced.

### 4.2. Examples of Drug Repositioning in Dermatology

Many instances of drug repositioning have been used to treat dermatological disorders (Table 1). Initially, doxepin was developed as a tricyclic antidepressant, which works by inhibiting serotonin and norepinephrine reuptake. Currently, because of the advancements in several different prescription medications in the psychiatric field, doxepin is seldom used for the treatment of depression. Instead, pathways involved in histamine and muscarinic receptor signaling have contributed to the treatment of skin disorders including chronic urticaria or pruritus [17].

Finasteride is a medication that was initially formulated to treat benign prostatic hyperplasia. Finasteride inhibits 5α-reductase, inhibiting testosterone conversion to dihydrotestosterone (DHT). Low doses of finasteride have been used to treat hair loss in men as this process is often associated with androgenic alopecia [18].

Minoxidil was originally developed for the treatment of hypertension as a potent peripheral vasodilator but was found to have hair growth as one of the clinical side effects. Minoxidil has also been developed for the treatment of androgenic alopecia [19].

A representative example of drug repositioning in pigmentation disorders is tranexamic acid. Tranexamic acid, a plasmin inhibitor, has been used as an anticoagulant in the past but is now used as a melasma-whitening agent [20]. Chemical peels in combination with other drugs are used for limited forms, and oral tranexamic acid may be used in the treatment of refractory melasma after screening for thromboembolism [21]. In a previous study, tranexamic acid was found to inhibit plasminogen binding to keratinocytes, preventing UV-induced pigmentation and reducing the tyrosinase activity of melanocytes. The inhibition of melanogenesis in melanocytes by interrupting the connection between melanocytes and keratinocytes has been suggested [22].

Many research groups have looked into the mechanism of drug repositioning related to the inhibition of tyrosinase activity. Ethionamide, a secondary anti-tuberculosis agent used to treat multidrug-resistant tuberculosis, shares a chemical similarity with tyrosinase inhibitors, and reduces melanin content [23].

Thiopurine drugs offer other examples of drug repositioning as tyrosinase inhibitors. Thioguanine, a drug used to treat acute leukemia, has been shown to decrease melanin content by inhibiting tyrosinase activity [24].

Nelfinavir, an HIV1-protease inhibitor, was identified as a potent suppressor of MITF expression in a drug repositioning study relevant to the MAPK pathway. Nelfinavir improves the approach of targeted melanoma therapy by sensitizing *BRAF* and *NRAS* mutant melanomas to MAPK pathway inhibitors [25].

We present an overview of several studies on the regulation of melanogenesis through drug repositioning in Table 2.

## 5. Research Results to Develop Pigmentation-Regulating Agents by Drug Repositioning

### 5.1. Induction of Pigmentation by a Small Molecule Tyrosine Kinase Inhibitor Nilotinib

Nilotinib is a drug approved by the FDA to treat chronic myeloid leukemia (CML). *BCR-ABL* tyrosine kinase is a particular inhibitor developed to resolve the issue of intolerance of and resistance to imatinib [30,31]. Originally, nilotinib was developed for CML, but other mechanisms were also identified. For example, in a myoblast cell line, nilotinib inactivated *p38* MAPK [32]. Furthermore, nilotinib prevented the kinase activity of the discoidin domain receptor (DDR) in metastatic colorectal cancer cells [33]. Such data suggest that nilotinib has more target points than the original BCR-ABL tyrosine kinase.

In our research on the effect of nilotinib on pigmentation, we found that in melanoma cells, nilotinib induced pigmentation [34]. When nilotinib was combined with melanoma cells, the pigmentation of melanoma cells was boosted. Melanin content and tyrosinase activity were greatly increased by nilotinib in both dose- and time-dependent manners. Nilotinib improved the expression of genes related to pigmentation including MITF, tyrosinase, and TRP-1. Nilotinib also demonstrated large changes in the amounts of MITF, tyrosinase, and TRP-1 protein. Nilotinib greatly lowered AKT phosphorylation as a result of its effect on signaling pathways in melanoma cells. Since the AKT signaling pathway negatively affects the process of pigmentation [35], the inhibition of AKT phosphorylation by nilotinib is considered to be an essential process for pigmentation induction. In addition, nilotinib has been correlated with the cAMP/PKA signaling pathway, which positively controls the mechanism of pigmentation. The effect of nilotinib on cAMP/PKA signaling was shown by measuring CREB phosphorylation, a downstream signal of cAMP/PKA, resulting in increased CREB phosphorylation. When we treated melanoma cells pretreated with PKA inhibitor H89 with nilotinib, CREB phosphorylation was significantly disrupted, sequentially blocking PKA signaling. In conjunction with these findings, nilotinib-induced pigmentation was prevented by pretreatment with H89. Therefore, the activation of the cAMP/PKA signaling pathway by nilotinib is proposed as one of the mechanisms essential for pigmentation induction.

**Figure 3:**
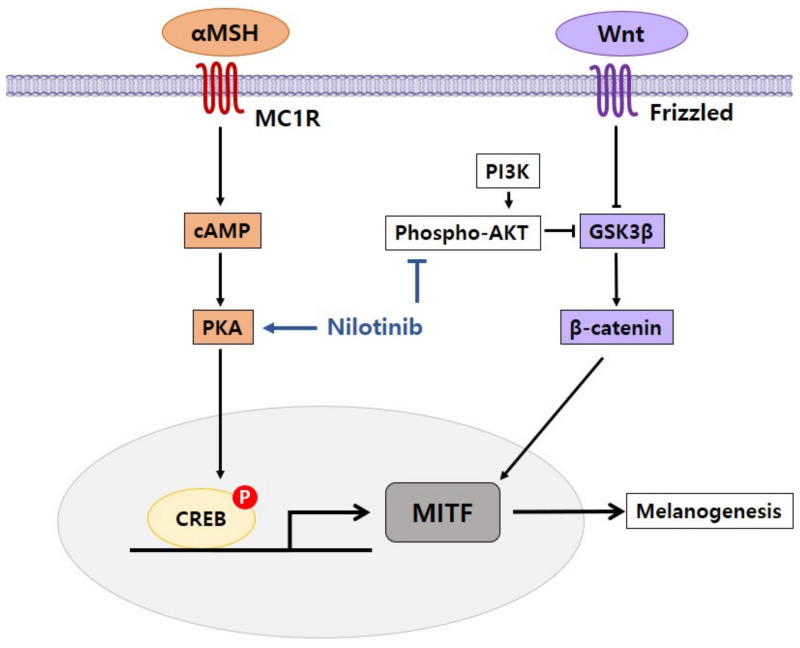
Proposed mechanism of nilotinib in the regulation of pigmentation. Nilotinib enhances melanogenesis by inhibiting phospho-AKT and activating cAMP/PKA.

In our study, we observed that nilotinib had the effect of controlling signaling pathways involved in pigmentation processes such as AKT and cAMP/PKA to enhance pigmentation in melanoma cells (Figure 3). Our research indicates that nilotinib could be used as an effective therapy for hypopigmentation disorders.

### 5.2. Sorafenib Induces Pigmentation via the Regulation of β-Catenin Signaling Pathway in Melanoma Cells

Sorafenib is a therapeutic agent approved by the FDA that can be used to treat a number of cancers, including unresectable hepatocellular carcinoma (HCC) and advanced renal cell carcinoma (RCC) [36]. Although it was originally conceived as an inhibitor of *Raf1*, there is some evidence that sorafenib has several other target proteins. For example, by targeting vascular endothelial growth factor (VEGF) receptors and platelet-derived growth factor (PDGF) receptors, sorafenib prevents angiogenesis [37].

Reinforcing the concept of several target proteins, our study found that by controlling many signal pathways, sorafenib may promote pigmentation in melanoma cells [38]. The pigmentation of melanoma cells was improved when sorafenib was combined with melanoma cells. Sorafenib also increased the melanin content and tyrosinase activity in both dose- and time-dependent manners. Sorafenib improved the amount of MITF, tyrosinase, and TRP-1 mRNA and protein by its effect on the expression of genes involved in pigmentation. Sorafenib significantly decreased the phosphorylation of AKT and ERK, demonstrating its effects on signaling pathways. These findings suggest that the inhibition of the AKT and ERK pathways is a sorafenib-related pigmentation process. The effect of sorafenib on the Wnt/β-catenin signaling pathway was also analyzed. Sorafenib dramatically increased the amount of active β-catenin and total β-catenin, suggesting that the intracellular β-catenin signaling pathway was activated by sorafenib. The effect of β-catenin on GSK3β was also assessed since the amount of β-catenin is controlled by GSK3β. Sorafenib was shown to decrease the phosphorylation of S9 of GSK3β, whereas it increased the phosphorylation of Y216 of GSK3β, resulting in the increased enzymatic activity of GSK3β. These findings suggest that sorafenib stimulated β-catenin signaling by controlling the phosphorylation of GSK3β in the pigmentation process.

**Figure 4:**
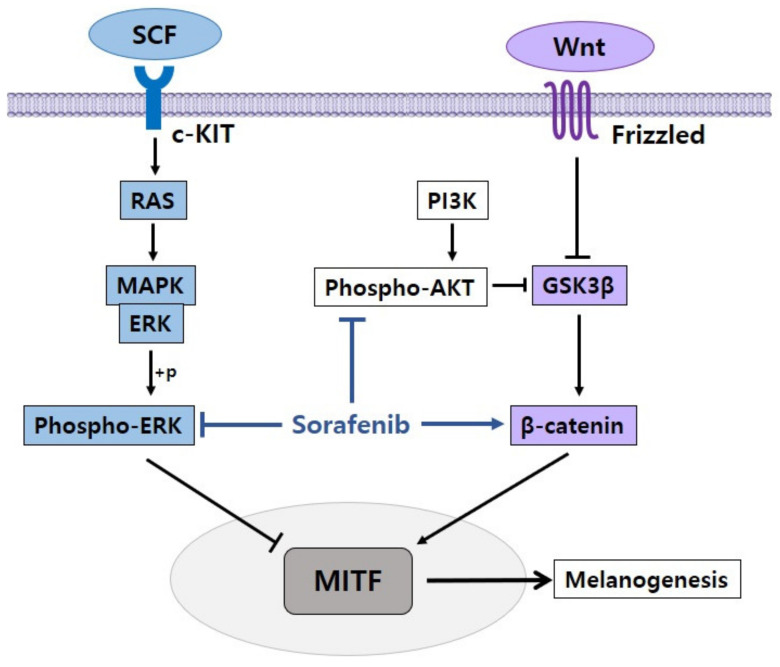
Proposed mechanism of sorafenib in the regulation of pigmentation. Sorafenib enhances melanogenesis by inhibiting phospho-AKT and phospho-ERK and activating β-catenin.

In our research, we found that sorafenib induced pigmentation in melanoma cells by inhibiting the AKT and ERK signaling pathways and regulating the β-catenin signaling pathway (Figure 4). Furthermore, the prospect of sorafenib as a repositioning agent that can be utilized in the treatment of hypopigmentation disorders was confirmed.

### 5.3. Wnt/β-Catenin Signaling Inhibitor ICG-001 Enhances Pigmentation of Cultured Melanoma Cells

ICG-001, which targets Wnt/β-catenin signaling, was initially designed to treat colorectal cancer by binding to CBP and preventing the connection between β-catenin and CBP [39]. As described earlier, the importance of the Wnt/β-catenin signaling pathway related to melanocyte formation and differentiation is well known. Thus, the Wnt/β-catenin signaling pathway may be a good target for the production of pigmentation-regulating agents.

Several Wnt/β-catenin signaling inhibitors including tankyrase, porcupine, and CBP have been tested in our research [40]. Contrary to predictions, certain chemicals either failed to decrease melanoma cell pigmentation or demonstrated no substantial decrease in pigmentation. Instead, ICG-001, one of the Wnt/β-catenin signaling inhibitors, demonstrated an improvement in melanoma cell pigmentation, along with an increase in melanin content and tyrosinase activity in dose- and time-dependent manners. The effect of the pigmentation caused by ICG-001 was replicable in another melanoma cell line. In terms of gene expression, ICG-001 increased the mRNA and protein levels of genes including MITF, tyrosinase, and TRP-1. CREB phosphorylation was induced by ICG-001 with an effect on the intracellular signaling pathways. The amount of intracellular cAMP measured after treatment with ICG-001 also increased early, suggesting that ICG-001 triggered the PKA signaling pathway. In contrast, the phosphorylation of AKT and ERK was not influenced by ICG-001. CREB phosphorylation by ICG-001 was markedly inhibited in melanoma cells pretreated with the PKA inhibitor H89, resulting in the inhibition of pigmentation and tyrosinase activity. Based on these findings, the pigment-affecting potential of ICG-001 in melanoma cells is believed to be correlated with the activation of the PKA pathway.

In our research, we observed that many inhibitors of Wnt/β-catenin signaling did not alter pigmentation in melanoma cells, whereas ICG-001, an effective inhibitor of Wnt/-β-catenin signaling, induced pigmentation in melanoma cells by triggering the PKA signaling pathway. Our results suggest that PKA signaling beyond Wnt/β-catenin signaling is essential in the pigmentation mechanism itself. Furthermore, ICG-001 may be an alternative substance for the treatment of hypopigmentation disorders.

### 5.4. Inhibitory Effect of 5-Iodotubercidin on Pigmentation

5-Iodotubercidin is an adenosine kinase inhibitor that has been identified as an activator of tumor suppressor p53 and has demonstrated anti-tumor efficacy by decreasing the size of tumors. 5-Iodotubercidin has been investigated as a possible genotoxic drug in chemotherapy since tumor suppressor p53 has been identified as an important target in the production of anti-cancer drugs [41].

We showed an inhibitory effect of 5-iodotubercidin on the pigmentation process in melanoma cells [42]. When we treated melanoma cells with 5-iodotubercidin, pigmentation was markedly decreased. Melanin content and tyrosinase activity were both substantially decreased by 5-iodotubercidin in a dose-dependent manner. With respect to gene expression for pigmentation, 5-iodotubercidin reduced the protein level of MITF, tyrosinase, and TRP-1. In terms of the effects on signaling pathways, 5-iodotubercidin diminished CREB phosphorylation, whereas it increased AKT and ERK phosphorylation. These findings suggest that 5-iodotubercidin is related to the inhibition of melanogenesis by modulating pathways including PKA and AKT/ERK signaling.

**Figure 5:**
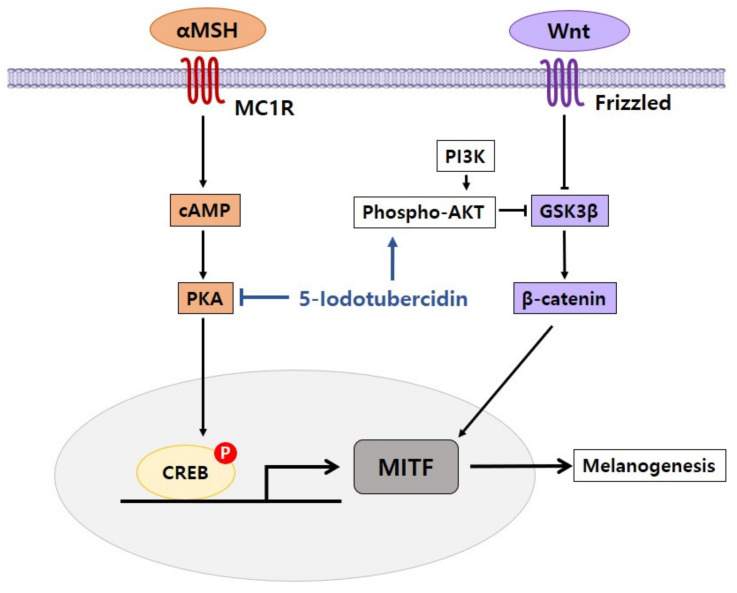
Proposed mechanism of 5-iodotubercidin in inhibiting effects of pigmentation. 5-Iodotubercidin inhibits melanogenesis by inhibiting cAMP/PKA and activating phospho-AKT.

Our research demonstrated that 5-iodotubercidin had an inhibitory effect on melanogenesis by regulating many signaling pathways related to pigmentation (Figure 5). 5-Iodotubercidin is intended to be used as one of the compounds for the production of depigmentation and skin-lightening agents.

## 6. Conclusions

Drug repositioning is an effective method to identify new drug uses by leveraging the effects of medications that have already been approved. Not only does this minimize the time and expense needed for new drug production, but since their efficacy has already been demonstrated, it also has a high likelihood of showing effectiveness in drug development. Our research team has researched the pathways involved in pigmentation modulation by treating melanocytes with particular substances or medications (Table 3). Based on the findings of our previous studies, we have been conducting research aimed at improving pigmentation-regulating agents that can potentially be used by drug repositioning to treat pigmentation disorders.

By influencing the pathways linked to pigmentation, all four forms, nilotinib, sorafenib, ICG-001, and 5-iodotubercidin, can be used to regulate pigmentation. In particular, the first three compounds or medications have been found to play a role in encouraging pigmentation, with the fourth compound serving to suppress pigmentation. It is also proposed that, for pigmentation disorders, these compounds or medications may be used as therapeutic or cosmetic agents.

## Figures and Tables

**Table 1:** Examples of drug repositioning in dermatology.

Generic	Original Indication	New Indication	Reference
Doxepin	Depressive disorder	Chronic urticaria, Pruritus	[17]
Finasteride	Benign prostatic hyperplasia	Androgenic alopecia	[18]
Minoxidil	Hypertension	Androgenic alopecia	[19]
Tranexamic acid	Anticoagulant	Melasma	[20,21,22]
Ethionamide	Tuberculosis	Anti-melanogenesis	[23]
Thiopurine	Acute leukemia	Anti-melanogenesis	[24]
Nelfinavir	HIV1-protease inhibitor	Melanoma	[25]
Spironolactone	Hypertension	Androgenic alopecia	[26]
Dapsone	Leprosy	Dermatitis herpetiformis, Acne vulgaris	[27]

**Table 2:** An overview of previous studies on the regulation of melanogenesis by drug repositioning.

Author	Year of Publication	Results
Choi J et al. [23].	2015	- Ethionamide (anti-tuberculosis agent) exhibit potent inhibition of tyrosinase, resulting in reducing melanin content.
Smith MP et al. [25].	2016	- Nelfinavir (HIV1-protease inhibitor) is repositioned as a suppressor of MITF expression and sensitizes *BRAF* and *NRAS* mutant melanomas to MAPK pathway inhibitors.
Choi J et al. [24].	2017	- Thiopurine drugs (anti-cancer agent) were repositioned as tyrosinase inhibitor, resulting in reducing melanin content.
Ullah S et al. [28].	2019	- Cinnamamide analogues have tyrosinase inhibition and anti-melanin generation effect.
Goenka S et al. [29].	2020	- Auranofin (anti-rheumatism agent) is identified as a depigmenting agent for hyperpigmentation disorders and adjuvant for melanoma therapeutics.

**Table 3:** Summary of research results on substances and drugs used to develop pigmentation-regulating agents by drug repositioning.

Substances or Drugs	Pigmentation		Pigmentation— Related Gene Expression	Signal Pathway	Original Indication
	Melanin Content	Tyrosinase Activity			
Nilotinib	↑	↑	↑	AKT↓ cAMP/PKA↑	CML
Sorafenib	↑	↑	↑	AKT/ERK↓ Wnt/β-catenin↑	HCC, RCC
ICG-001	↑	↑	↑	cAMP/PKA↑	Colorectal cancer
5-Iodotubercidin	↓	↓	↓	AKT/ERK↑ cAMP/PKA↓	Anti-cancer drug

## Data Availability

Data are contained within the article.

## Abbreviations

α-MSH	α-melanocyte-stimulating hormone
ACh	acetylcholine
AChE	acetylcholinesterase
AChR	acetylcholine receptor
AKT	protein kinase B
APC	adenomatous polyposis coli
cAMP	cyclic adenosine monophosphate
c-KIT	tyrosine kinase receptor
CBP	CREB-binding protein
CML	chronic myeloid leukemia
CREB	cAMP response element-binding protein
DDR	discoidin domain receptor
DHT	dihydrotestosterone
ERK	extracellular signal-regulated protein kinase
GSK3β	glycogen synthase kinase 3β
HCC	hepatocellular carcinoma
MAPK	mitogen-activated protein kinase
MC1R	melanocortin 1 receptor
MITF	microphthalmia-associated transcription factor
PDGF	platelet-derived growth factor
PI3K	phosphatidylinositol 3-kinase
PKA	protein kinase A
RCC	renal cell carcinoma
S9	serine 9
SCF	stem cell factor
TRPs	tyrosinase-related proteins
VEGF	vascular endothelial growth factor
Wnt	wingless-related integration site
Y216	tyrosine 216
